# Subpixelic Measurement of Large 1D Displacements: Principle, Processing Algorithms, Performances and Software

**DOI:** 10.3390/s140305056

**Published:** 2014-03-12

**Authors:** Valérian Guelpa, Guillaume J. Laurent, Patrick Sandoz, July Galeano Zea, Cédric Clévy

**Affiliations:** 1 Automation and Micro-Mechatronics Systems Department, FEMTO-ST Institute, UMR CNRS 6174, ENSMM, Université de Franche-Comté, 25000 Besançon, France; E-Mails: valerian.guelpa@femto-st.fr (V.G.); cclevy@femto-st.fr (C.C.); 2 Applied Mechanics Department, FEMTO-ST Institute, UMR CNRS 6174, ENSMM, Université de Franche-Comté, 25000 Besançon, France; E-Mail: psandoz@univ-fcomte.fr; 3 Instituto Tecnológico Metropolitano, Grupo de Materiales Avanzados y Energía, 05001 Medellin, Colombia; E-Mail: julygaleano@itm.edu.co

**Keywords:** displacement sensing, subpixelic measurement, nanometric precision, extended range-to-resolution ratio, real-time image processing, twin-grid pattern, direct phase computation

## Abstract

This paper presents a visual measurement method able to sense 1D rigid body displacements with very high resolutions, large ranges and high processing rates. Sub-pixelic resolution is obtained thanks to a structured pattern placed on the target. The pattern is made of twin periodic grids with slightly different periods. The periodic frames are suited for Fourier-like phase calculations—leading to high resolution—while the period difference allows the removal of phase ambiguity and thus a high range-to-resolution ratio. The paper presents the measurement principle as well as the processing algorithms (source files are provided as supplementary materials). The theoretical and experimental performances are also discussed. The processing time is around 3 μs for a line of 780 pixels, which means that the measurement rate is mostly limited by the image acquisition frame rate. A 3-*σ* repeatability of 5 nm is experimentally demonstrated which has to be compared with the 168 μm measurement range.

## 1. Introduction

Numerous scientific fields such as micromechatronics and instrumentation for micro-, nano- and bio-technologies require a high accuracy in the tracking of objects as well as in the manipulation of actuators and stages. For such position and displacement control purposes at the microscopic scale, vision-based approaches are often found to be the best suited methods. The resolution of the camera combined with the imaging magnification is then an important performance limitation parameter with a general consequence: the higher the accuracy, the lower the range. Numerous sub-pixel motion detection algorithms have been proposed to relax this kind of trade-off. A common practice is the phase correlation method. To achieve the translation estimation at sub-pixel level, some researchers use peak interpolation methods in the spatial domain [1,2]. Another approach is to oversample images, but this method increases the computing load dramatically. Authors like Douglas [3] and Gao *et al.* [4] use a direct measurement in the frequency domain and, through combination with statistical methods, obtain respectively 3-*ó* precisions around 5 nm and 3 nm.

High accuracy can also be obtained through phase computations by means of Fourier-like processing applied to a periodic grid. Despite phase-to-displacement conversion providing high measurement resolution, a main drawback comes from the 2*kπ* phase uncertainty due to the definition domain of the inverse tangent function, as observed for instance in Yamahata's case [5]. The measurement range of these methods is basically limited to a single period of the pattern. A common solution to avoid this phase ambiguity consists in using a pseudo-periodic pattern that embeds some kind of binary code [6–9]. The visual position detection works then over larger measurement ranges. However, from a computational point of view, these methods are time-consuming and often incompatible with real-time applications.

Another way to overcome the 2*kπ* phase ambiguity consists in using two slightly different reference periods [10,11]. The phase mismatch observed between the two independent phase computations allows the removal of the phase ambiguity and the measurement range is thus extended by a factor from 5× to 50× at least, determined by the actual period difference and limited by the detection signal-to-noise ratio. This principle has been applied to different measurement purposes [12–14].

This paper presents the implementation of this principle for the visual measurement of 1D rigid body displacements with very high resolutions, large ranges and high processing rates. The method is based on a pattern made of twin periodic grids that allows for phase calculations while the period difference is used to extent the measurement range.

The next section introduces the measurement principle in detail. Section 3 presents the processing algorithms (source files are provided as supplementary materials). Afterwards, we discuss the theoretical capabilities of the method as well as the experimental results obtained.

## 2. Principle: Displacement Measurement from Twin Stripe sets with Slightly Different Periods

The phase-to-displacement relationship is widely known. If an object *O* is shifted in a space *X* from an initial position *O_i_*(*x*) to a final position *O_f_*(*x*), the displacement can be written mathematically as a convolution product:

(1) $$O_{f}(x)=O_{i}(x)*\delta (x-\Delta )$$

where * stands for the convolution product, *δ*(*x*) for the Dirac impulse distribution and Δ for the displacement value. After Fourier transformation we obtain a simple product in the frequency domain:

(2) $${\hat{O}}_{f}(\nu )={\hat{O}}_{i}(\nu )\cdot e^{j2\pi \nu \Delta }$$

where *Ô_i_* and *Ô_f_* are the Fourier transforms of *O_i_*(*x*) and *O_f_*(*x*) respectively, *ν* and *x* are reciprocal variables and *j* the complex number *j*^2^ = −1. Equation (2) shows that in the frequency domain, the object displacement induces only a phase shift equal to 2*πν*Δ, a direct consequence of the Fourier transform.

Thanks to this linear relationship, the object displacement is encoded in the phase of the Fourier transform and different approaches have been proposed for the measurement of displacements through this phase term. They differ mainly in the number of considered spectral components. If the whole Fourier spectrum is considered, an unambiguous value of Δ can theoretically be retrieved. However, the right combination of phase constants 2*k_ν_π* has to be found and this task may require iterative algorithms [15] or calibrated actuators as in its application to surface profilometry [16]. Such an extended phase processing is time-consuming and not suited for real time applications. Faster approaches consider only one or a few spectral components. In this case the displacements retrieved may be subject to phase ambiguities that limit the actual unambiguous measurement range as described below. However there are numerous applications in which this limitation is not critical; provided that the unambiguous measurement range can be matched with practical requirements.

### 2.1. Ambiguous Displacement Measurement from a Single Periodic Pattern

**Figure 1.**
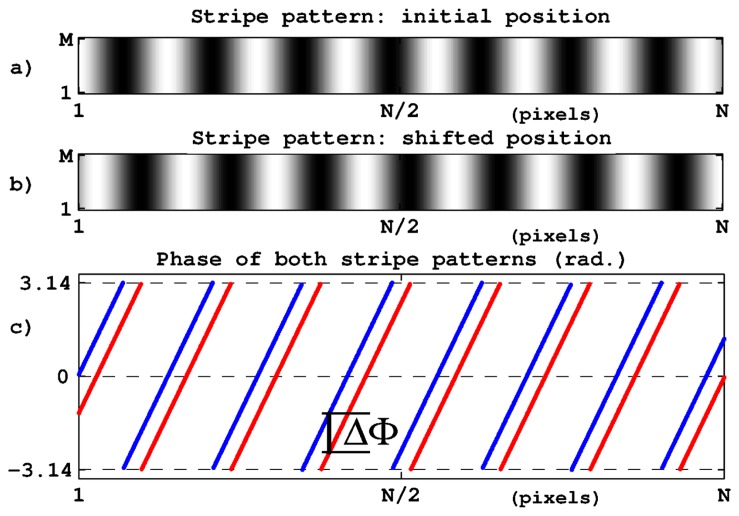
Correspondence between lateral position and phase of a sinusoidal pattern. (**a**) stripe set before displacement; (**b**) after displacement; (**c**) wrapped phase for both positions.

In computer vision, the target displacements are retrieved through the processing of images captured by a static camera observing the moving object. The simplest way to apply phase computation to this task consists in associating some kind of periodic pattern to the target and thus to get periodically structured images for processing. This is illustrated in Figure 1a in which the stripe set corresponds to the target image recorded in its initial position. Figure 1b shows the image recorded after a target displacement in the direction perpendicular to the stripes. The target displacement appears clearly through the stripe position and, as explained in Equation (2), it induces a phase shift ΔΦ between the two stripe sets as represented in Figure 1c. The target displacement can then be determined by:

(3) $$\Delta =\frac{\Delta \Phi \cdot P}{2\pi }+kP$$

where *P* is the stripe period and *k* is an unknown integer standing for an entire number of stripe periods (We notice in Equation (3) that the vision system magnification does not need to be known since the actual period *P* of the target stripes serves as a dimensional reference, provided that the period is known or measured with sufficient accuracy.). Indeed due to the stripe periodicity, the displacement value is obtained modulo *P* since different positions distant from an entire number of periods produce indistinguishable images. This ambiguity is due to the definition domain ]−*π*, *π*] of the inverse tangent function. It restricts the unambiguous measurement range to a single stripe period. The measurement range and measurement resolution are thus dependent on each other and the number of resolved positions is equal to *K* = 2*π*/*δ*Φ, where *δ*Φ is the resolution of the phase determination. The only adjustment parameter is *P* that affects range and resolution in inverse proportions and thus does not affect the range-to-resolution ratio. The latter can only be improved through the phase computation performances. In practice, image digitizing, electronic noise and environmental disturbances form irreducible noise sources. As explained below, the use of a second stripe period is an alternative way to extend the measurement range without decreasing the resolution.

### 2.2. Removal of Phase Ambiguities from Slightly Different Periods

**Figure 2.**
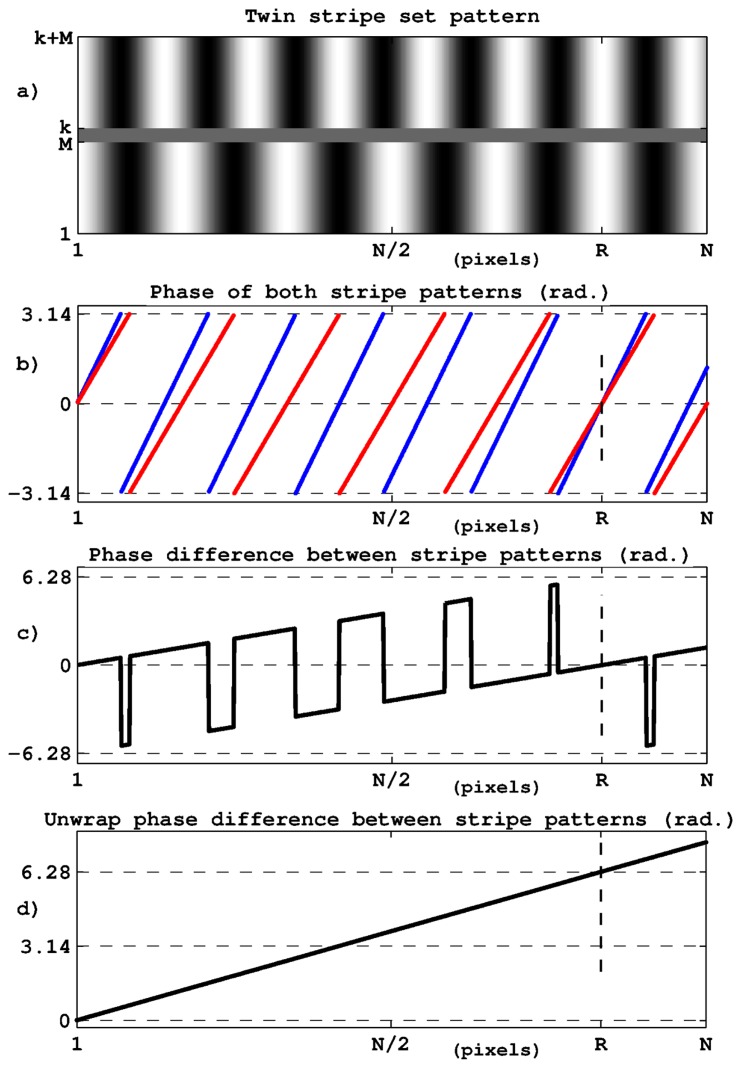
Extended unambiguous range by means of two stripe sets with different periods. (**a**) twin stripe sets; (**b**) phase of both stripe sets; (**c**) phase difference between stripe sets; (**d**) unwrapped phase difference. Position ‘R’ corresponds to phase coincidence, it marks the new ambiguity range obtained.

The use of a second stripe set with a slightly different period provides complementary and independent phase data that can be used for the removal of phase ambiguities. The principle is illustrated in Figure 2. Figure 2a shows the pattern image with two stripe sets with different periods. As illustrated in Figure 2b, the resulting phases present different combinations from one stripe to the next one. We only obtain perfect data reproduction at pixel *R*; *i.e.*, when the stripes have the same position with respect to each other as at the left side of the image. Thanks to the progressive mismatch between the two stripe sets, phase ambiguities can be removed and the unambiguous range switches from a single period to a new value Λ given by:

(4) $$\Lambda =\frac{P_{1}\cdot P_{2}}{\left|P_{1}-P_{2}\right|}$$

where *P***_1_** and *P***_2_** are the stripe set periods (The phase coincidence between the two stripe sets does not necessarily correspond to an intensity maximum but may occur at any location depending on the combination of periods. For instance, with periods of *P*_1_ = 6 and *P*_2_ = 10, the equivalent period is Λ = 15 whereas the perfect reproduction of the stripe sets is only obtained at 30.).

From a computational point of view, the phase relative to the synthetic period Λ is simply given by the subtraction between the two elementary phases (Figure 2c) after phase unwrapping as represented in Figure 2d. The conversion from phase to displacement is still given by Equation (3) in which *P* is replaced by Λ. In fact, because of the phase subtraction, noise is magnified in the same proportion as the unambiguous range and a supplementary step is necessary to improve the range-to-resolution ratio. This step consists in using synthetic data to determine the correct 2*kπ* constant to apply to the phase shift observed for either stripe set. Equation (3) thus becomes:

(5) $$\Delta =\frac{\Delta \Phi_{1}\cdot P_{1}}{2\pi }+k_{1}P_{1}+k_{x}\Lambda$$

in which ΔΦ_1_ is the phase shift for the chosen stripe set, *k*_1_ the number of periods derived from the synthetic phase and *k_x_* an unknown number of periods Λ that represents the new ambiguity range. The range-to-resolution improvement resulting from this twin period method is equal to Λ/*P*_1_.

Theoretically, Λ can be chosen as large as we want (Equation (4)) through the choice of *P*_1_ and *P*_2_. We notice however that large values of Λ are obtained with small period differences. In such cases the effects of noise are significantly enlarged and, at some level, the determination of *k*_1_ (Equation (5)) fails and errors are introduced. In practice, the measurement range enlargement has to be matched with the signal-to-noise ratio and with the phase resolution *δ*Φ achieved, thus optimizing the trade-off between measurement range and robustness.

In Figure 2b the phase of both stripe sets are represented as a function of the pixel index from a single pattern image. This does not correspond exactly to the case of displacement measurement in which an image sequence has to be processed to retrieve a single phase value per frame. In fact in Figure 2b, the phase combinations observed between pixels 1 to *R* describe all possible phase values that can be associated with any image pixel; for instance that at the center of the image. This is indeed the information representative of the target position or displacement. The aim of the signal processing is thus to determine accurately the phase at the central image pixel for the spatial frequency of each stripe set. In this task, the whole image width is involved in a Fourier-like phase computation as described in Section 3.

## 3. Processing Algorithms and Software Implementation

In the following, we present an implementation for 1D lateral displacement measurement in a micro-mechatronic context as well as the performances obtained. This principle and the software provided can however be applied to different purposes, especially at other dimensional scales.

**Figure 3.**
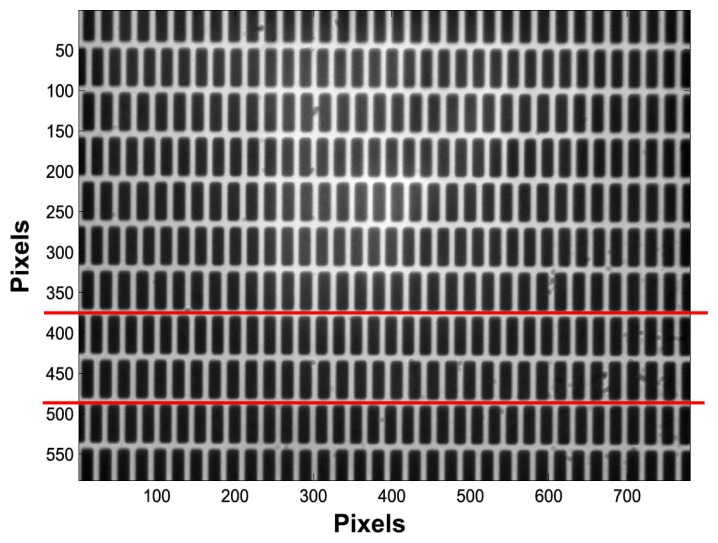
Experimentally recorded image of the twin stripe set pattern on the target.

We assume that a two-stripe pattern is attached to the target of interest in order to measure its displacement. Figure 3 gives an example of image to be processed as recorded experimentally using a 20× microscope lens. It is composed of a series of ruling grids where the 8 μm and 8.4 μm grids are distributed alternatively.

The determination of the phase associated with each image of this kind and representative of the target position at the recording instant of time assumes three successive steps described in the following subsections: extraction of intensity distributions from each stripe set; determination of the period in pixel of each stripe set; computation of the phase of the central pixel for both stripe sets

### 3.1. Determination of Image Lines associated with each Stripe Set

This preprocessing task aims to determine the image lines to be used for the phase computation for each stripe set. Since our aim is to measure only 1D displacements, the hardware is set in such a way that the stripes are perpendicular to the displacement and the camera lines are parallel to the stripes. With this setting, stripes move only in the horizontal direction of the recorded images and this task has to be performed only once. Since the used pattern is repetitive, there are several possibilities and for instance the stripes sets between the red lines can be chosen (*cf.* Figure 3). At this point, we can either use a single line for processing, for example located at the middle of the stripe set, or to average data by summing a few lines in order to increase the signal-to-noise ratio. Finally, for each recorded image, two intensity distributions *I*_1_(*l*) and *I*_2_(*l*) (*l pixel index*) are extracted and associated with stripe sets of period *P*_1_ and *P*_2_ respectively.

### 3.2. Determination of the Spatial Frequency of each Stripe Set

**Figure 4.**
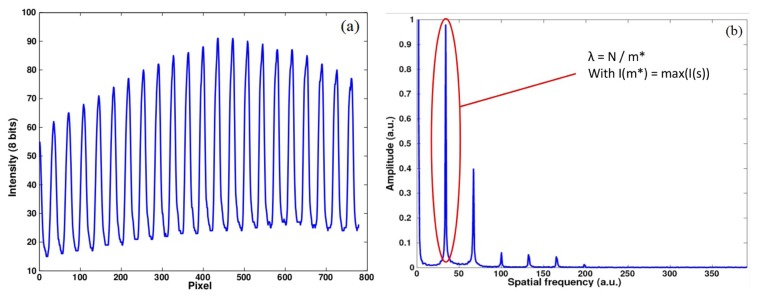
Example of intensity vector (**a**) of a real pattern and its Fourier transform (**b**).

This second step aims to determine the spatial frequency of each stripe set in the recorded images. This task has also to be performed only once since the imaging magnification is not affected by 1D lateral target displacements. Furthermore Equation (3) which allows the data conversion from phase to displacement requires that the working frequency remains the same allover the moving sequence. This task is performed by means of Discrete Fourier Transform (DFT) with a Gaussian apodization function (see Equation (9) in the next section). The DFT is defined by:

(6) $$\hat{I}(s)=\sum_{l=0}^{N-1}I(l)\cdot e^{-j2\pi ls/N}$$

where *I*(*l*) is the windowed intensity vector, *l* and *s* are reciprocal variables and *N* is the number of image pixels per line. Figure 4 presents an example of intensity distribution and of the magnitude of its DFT. We determine the stripe frequency by firstly locating the position *m* of the maximum DFT magnitude within a spectral interval [*a*, *b*]. This interval aims to remove background intensity and high frequency noise.

(7) $$m=\underset{s\in [a,b]}{\arg\max}|\hat{I}(s)|$$

Since the log of a Gaussian produces a parabola, we use a quadratic interpolation on the points around *m* in order to get a better localization of the maximum, such as:

(8) $$m^{*}=m-\frac{1}{2}\cdot \frac{\left(|\hat{I}(m)|-|\hat{I}(m+1)|)-(|\hat{I}(m)|-|\hat{I}(m-1)|\right)}{\left(|\hat{I}(m)|-|\hat{I}(m+1)|)+(|\hat{I}(m)|-|\hat{I}(m-1)|\right)}$$

The stripe period *P* in the pattern image is then given by $P=\frac{N}{m^{*}}$. This processing is applied twice; *i.e.*, once for each stripe set and thus periods *P*_1_ and *P*_2_ are obtained.

### 3.3. Computation of the Phase of the Central Pixel for both Stripe Sets

**Figure 5.**
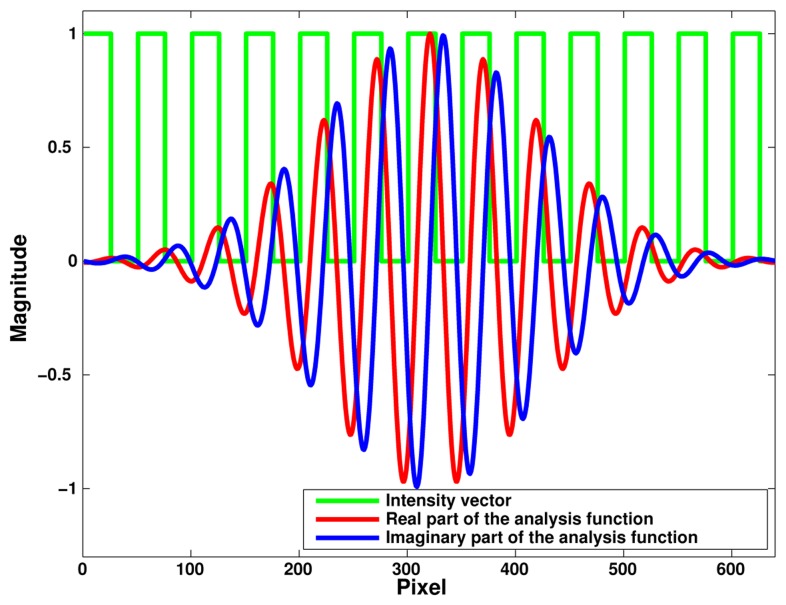
Analysis function used for phase computation.

Once the preprocessing described above is done, the aim of the phase computation is to determine the phase associated with both stripe sets as fast as possible. Then instead of performing a complete DFT we compute only the two phase terms of interest. For that purpose we use a complex analysis function *Z*_1_(*l*) defined by a Gaussian envelop and a periodic signal at the period of the stripe set (cf. Figure 5) (Theoretical accounts on the spectral effects of a so-called apodization window can be found in [17]):

(9) $$Z_{1}(l)=\exp\left(-{\left(\frac{l-N/2}{N/4.5}\right)}^{2}\right)\cdot \exp\left(\frac{-2j\pi (l-N/2)}{P_{1}}\right)$$

We notice that the analysis functions have also to be defined only once. The expected phase Φ_1_ is then given by the argument of the sum Σ_1_ over all pixels of the product of this windowed analysis function *Z*_1_(*l*) by the intensity distribution *I*_1_(*l*):

(10) $$\Sigma_{1}=\sum_{l=0}^{N-1}I_{1}(l)\cdot Z_{1}(l)$$

and

(11) $$\Phi_{1}=\tan^{-1}(𝔍𝔪(\Sigma_{1})/ℜ𝔢(\Sigma_{1}))$$

The phase Φ_2_ related to the second stripe set is computed in the same way by applying this procedure with *P*_2_ instead of *P*_1_ and *I*_2_(*l*) instead of *I*_1_(*l*). We then obtain the synthetic phase Φ related to the current image by: Φ = Φ_1_ − Φ_2_ which has to be unwrapped in the interval ]−*π*, *π*] by adding ±2*π* where necessary (*cf.* Figure 2).

### 3.4. Combination of Coarse and Accurate Data Leading to High Range-to-Resolution Ratio

For the current image, after the computation of phases Φ_1_, Φ_2_ and Φ, a coarse displacement Δ is given by Equation (3). To improve this coarse value and get a highly accurate displacement evaluation, the constants *k*_1_ of Equation (5) have to be determined from:

(12) $$\Delta =\Lambda \cdot \frac{\Phi }{2\pi }$$

and

(13) $$\Delta =P_{1}\cdot \frac{\Phi_{1}}{2\pi }+k_{1}P_{1}$$

We get

(14) $${\hat{k}}_{1}=\frac{\Lambda }{P_{1}}\cdot \frac{\Phi }{2\pi }-\frac{\Phi_{1}}{2\pi }$$

From a theoretical point of view, Equations (12) and (13) are equivalent. From an experimental point of view however, Equation (12) is more noisy than Equation (13). Because of that the value of *k̂*_1_ returned by Equation (14) is not exactly an entire number as it should be but only close to an integer. In practice the gain in resolution provided by considering the stripe set of period *P*_1_ (or *P*_2_) instead of that of the synthetic period Λ is obtained by rounding (*k*_1_ = *round*(*k̂*_1_) where *round*(*x*) returns the integer the closest to x) *k̂*_1_ to the closest integer *k*_1_. The high-resolution displacement measurement is finally provided by inserting the computed value of *k*_1_ in Equation (5) with *k_x_* = 0. This procedure leads to the final displacement value with a range-to-resolution ratio *RRR* of:

(15) $$RRR=\frac{\Lambda }{P_{1}}\cdot \frac{2\pi }{\delta \Phi }$$

## 4. Method Capabilities and Numerical Limitations

Without considerations for the material used, the ultimate capabilities of the method would be determined by the physics phenomena involved and, in our case, would imply quantum statistics of the light source of illumination and of photon conversion on the image sensor. Such fundamental sources of noise are responsible for extremely low error levels as expected for instance in gravitation wave detection interferometers. In the case of applications based on scientific grade instrumentation as aimed here, the ultimate performances are determined by material and environmental specifications. The most influential error sources in such visual experiments are thus due to digitizing and to the signal-to-noise ratio of the detected images. Environmental disturbances are also detrimental to high resolution displacement measurements and may be found to be the most restrictive parameter. They remain however independent of the intrinsic method capabilities and thus form extrinsic error sources.

**Figure 6.**
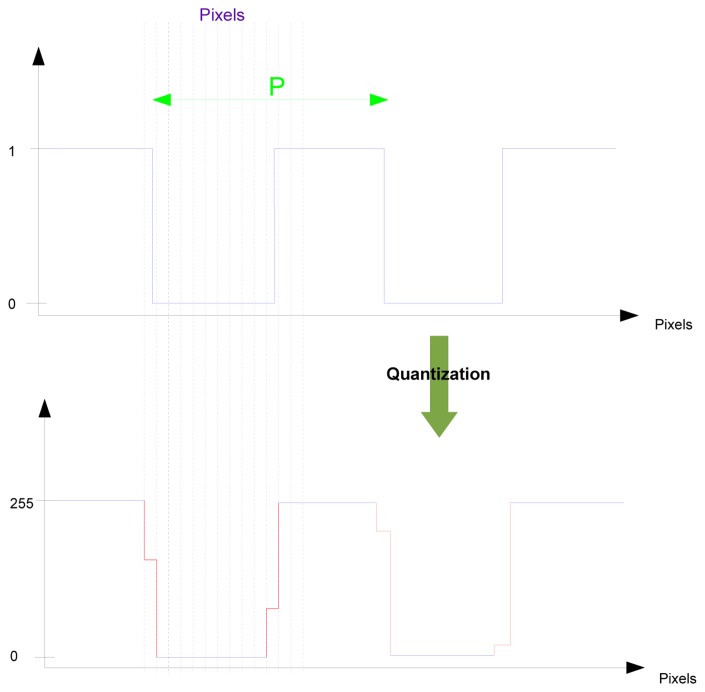
Illustration of the quantization principle applied to a perfect square grid; case of 8 bits sensor, with pixels encoded between 0 and 255.

Our method capabilities were evaluated through computations aimed to reconstruct the position of sets of computer generated images with chosen and perfectly known grid positions. Perfect square grids were used in the image design and light diffraction effects were not considered. This point is not crucial since a low pass filtering due to light diffraction would not affect the first harmonic of the square grid spectrum that is actually used in image processing (*cf.* Section 2). The square grids were digitized using area sampling with a fill factor equal to one emulating a perfect CCD sensor (see Figure 6).

**Figure 7.**
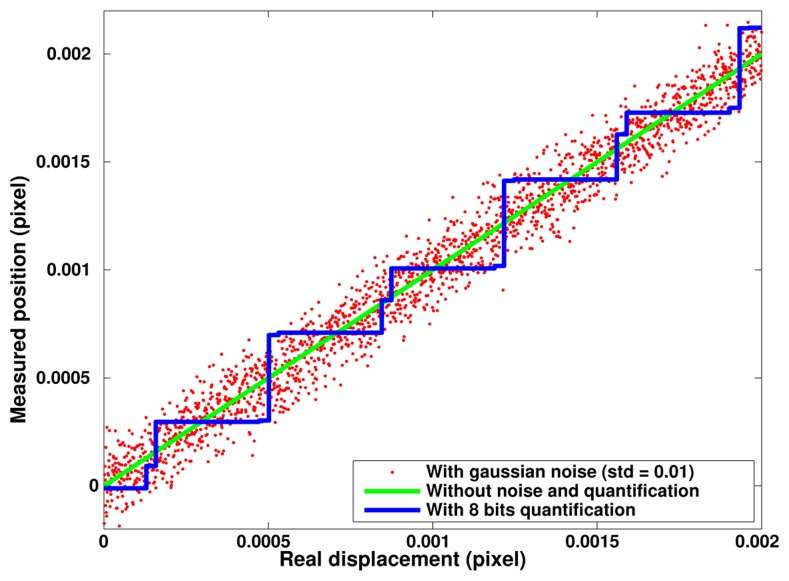
Simulated position reconstruction as a function of numeric noise (green), signal quantization (blue) and presence of noise (red); period of 51.123 pixels, computation step of 10^−6^ pixel.

Differences observed between the reconstructed positions and the ones used for image generation provide the actual method detection errors. Figure 7 presents the results of such computations for three different series of 1,000 images designed with an arbitrary period of 51.123 pixels and a elementary shift of 10^−6^ pixel between consecutive images. The green curve corresponds to the most favorable case involving only the computing noise. We can see that the expected linear displacement is perfectly reconstructed and in fact, the 10^−6^ pixel step is already too large to make the numeric noise effects visible. The blue curve corresponds to an image digitizing over 8 bit depth and we can see that the highest consecutive steps are of about 0.4 × 10^−3^ pixel (*i.e.*, close to 1/2^8^). The standard deviation of digitizing errors is of 10^−4^ pixel at this digitizing depth. The red curve was obtained without digitalization but by adding a gaussian noise to the generated grid with a standard deviation equal to 1% of the square amplitude. The resulting errors present a different distribution but approximately with the same peak-to-peak amplitude. The error standard deviation due to gaussian noise is equal to 9.6 × 10^−5^ pixel. These simulation results determine the best resolutions that can be expected experimentally with these parameters that correspond to typical specifications of usual image sensors.

**Figure 8.**
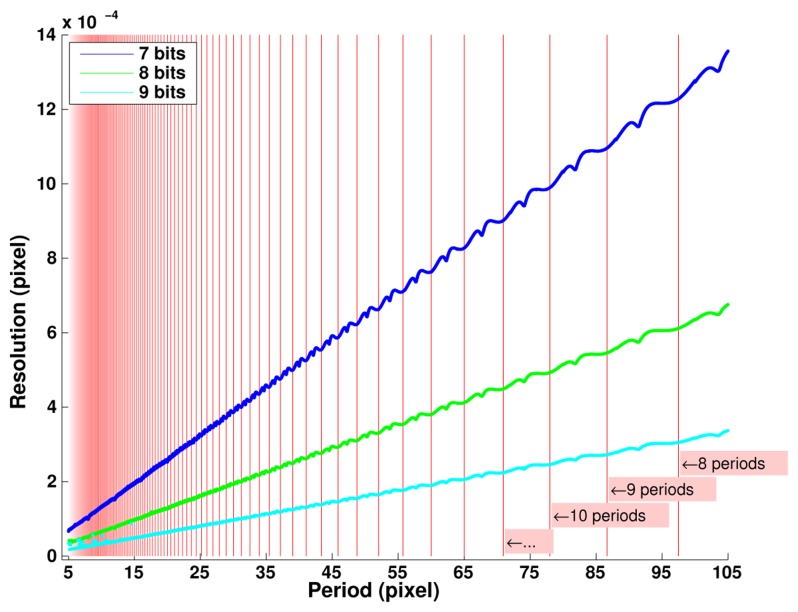
Ultimate resolution in pixels as a function of grid period and quantization depth (At each period, maximum error observed on 10^3^ positions shifted by 10^−6^ pixel).

Figure 8 explores in more details the effects of the digitizing depth and of the grid period value chosen for image generation. We observe a linear dependence of the error level with the grid period. One more bit of digitizing leads to a twofold decreasing in the resolution achieved. The non-linearities observed versus the period value are due to numeric effects depending on the ratio between the image pixel number *N* and the grid period. The best resolution is achieved when this ratio corresponds to an integer number—as indicated by the red vertical lines - which corresponds to the best representation of an infinite periodic signal as considered in continuous Fourier transforms.

**Figure 9.**
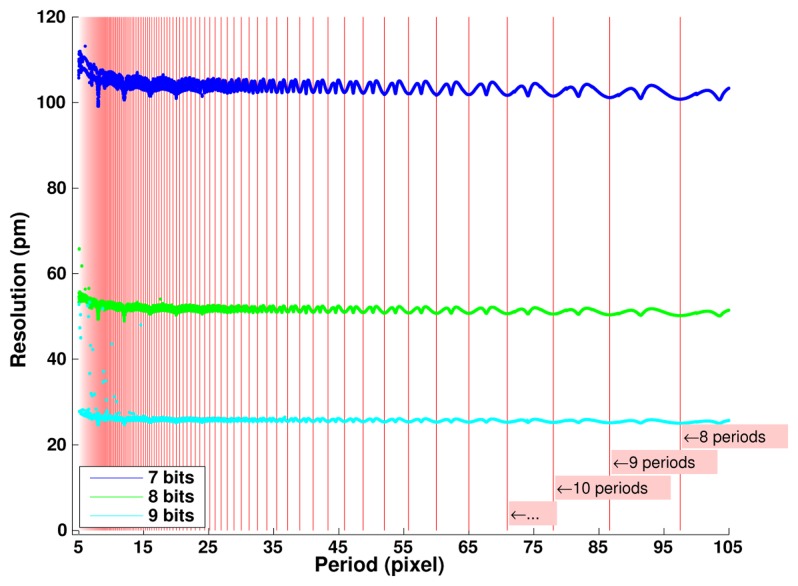
Ultimate resolution in picometers as a function of grid period and quantization depth (At each period, maximum error observed on 10^3^ positions shifted by 10^−6^ pixel, with a grid of period 8 μm).

If the period increase is due to an increase of the vision system magnification; *i.e.*, if the grid period remains the same; then after error conversion from pixels to actual distance, the resolution becomes independent of the period value as represented in Figure 9. In practice, magnification has to be matched with the actual period value to remain compatible with light diffraction limitations.

These numerical evaluations of the method capabilities can be used to match the setup design with the expected system performances. For instance, if sufficient free space has to be kept between the lens and the target, a larger grid period can be chosen to be suited with a low magnification objective and a large working distance. The resolution loss due to period increase can then be compensated by the use of a higher grade camera with improved signal-to-noise ratio and digitizing depth. Such an elementary period increase may also be chosen to enlarge the unambiguous measurement range as defined by Equation (4) as well as the range-to-resolution ratio.

## 5. Experimental Results and Performances

**Figure 10.**
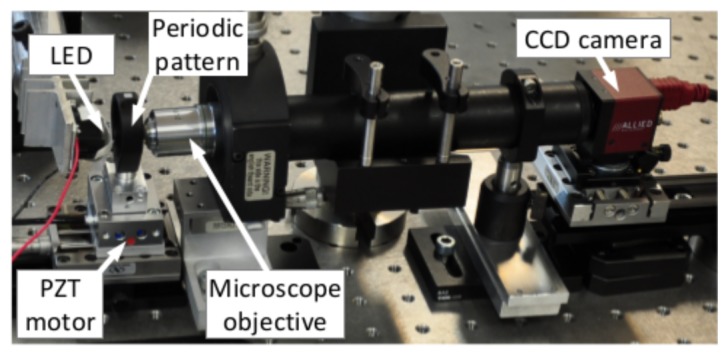
Experimental setup used for method demonstration.

Figure 10 presents a view of the experimental setup used. The grid patterns are realized by means of photolithography and clean room processes onto a transparent piece of glass. The periods of the realized stripe sets are respectively of 8 μm and 8.4 μm leading to a 168 μm unambiguous measurement range (or equivalent wavelength). The specimen obtained is back-illuminated by a white light emitting diode (Luxeon Star:0906LXHLND98) and imaged onto the camera sensor (Allied Vision Technology Guppy F-046, 780 × 582 pixels, cell size 8.3 μm, 8 bits quantization) through a 20× microscope lens (Edmund Optics Din 20×, N.A. 0.4). The grid pattern is mounted onto a servo-controlled piezoelectric linear stage (Physics Instruments P-753.1CD), allowing calibrated 1D displacements (repeatability 1 nm, linearity 0.03%). The internal capacitive sensor of the linear stage provides an independent position measurement used as a reference to evaluate the visual measurement performances (resolution 0.05 nm).

### 5.1. Quasi-Static Experiments

**Figure 11.**
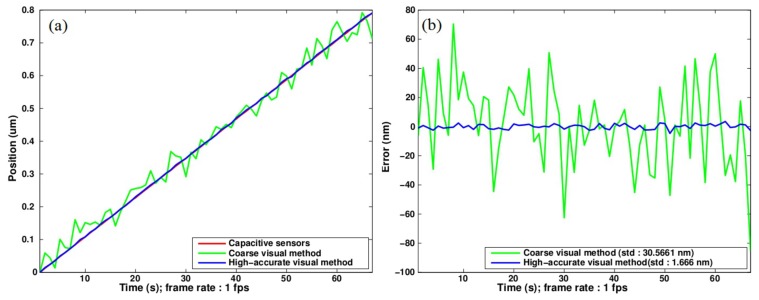
Measurement of linear displacement of the pattern. 780 × 580 pixels image. (**a**) Reconstructed positions; (**b**) Deviation from a straight line.

Figure 11a presents the experimental reconstruction of linear displacements as applied step by step to the grid pattern by means of the linear stage. A low acquisition rate of 1 fps is used because we target the static behavior of the system. The figure allows the comparison of the noise level attached respectively with the coarse and accurate displacement computations. The green curve is obtained through Equation (3) with *P* = Λ = 168 *μm* (Λ is the equivalent period defining the unambiguous measurement range). The blue curve is obtained from Equation (5) that is based on the small-sized period *P*_1_ after compensation for 2*π* phase jumps. The gain in resolution can be evaluated in Figure 11b that presents the deviation from a straight line. The standard deviation is reduced by about 20 times by switching from the equivalent period to the elementary one. On such a sub-micrometer excursion, the noise level quantified by the standard deviation is only 1.666 nm; leading to an estimated 3-*σ* precision of 5 nm. It could be compared to the 55 pm found as the optimal resolution of the method in these conditions, in Section 4. This gap is the result of environmental disturbances (thermal and mechanical): a 5 nm noise is normal for a macro-setup used in non-controlled atmosphere.

### 5.2. Dynamic (Real-Time) Experiments

**Figure 12.**
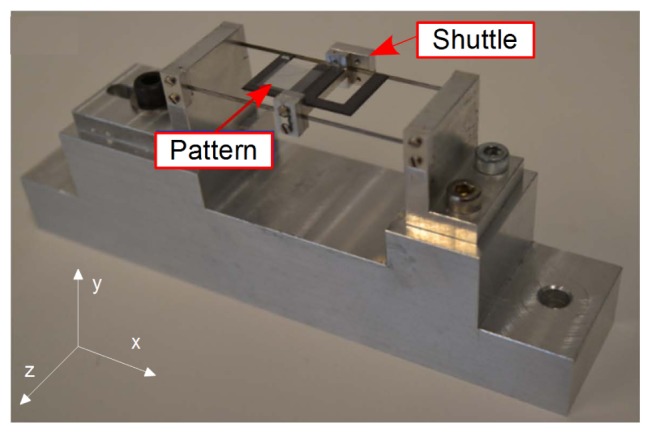
Shuttle used as target in real-time experiments.

**Figure 13.**
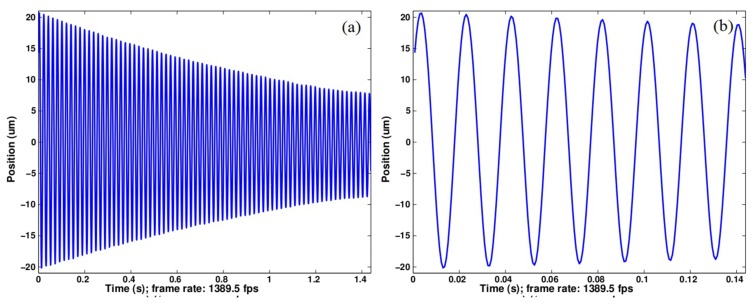
Reconstructed shuttle displacements in response to a starting pulse. (**a**) Exponentially decreasing vibration amplitude; (**b**) Zoom on the first cycles.

Finally the experimental setup was used to characterize the free oscillations of a compliant shuttle as shown in Figure 12. The moving part is attached to the static structure by means of two parallel and horizontal beams. Forces applied following the *Z* direction; *i.e.*, perpendicularly to the beams, result in 1D displacements of the shuttle. Such a device allows force-to-displacement transduction with a proportion ratio that is a function of the beam stiffness. A grid pattern was fixed onto the shuttle and observed by means of the vision system. The camera was replaced by a fast Firewire camera (Allied Vision Technology Pike F-032B). The exposure time was reduced to 18 μs and a region of interest of 320 × 26 pixels was selected in the image in order to increase the acquisition rate. A frame grabbing of 1389.5 fps was achieved with these parameters and a common computer (Intel Core2 Quad CPU Q9550 2.83 GHz, running under Windows 7). The C++ software developed (Provided as supplementary data to this paper.) was able to process the image flow in real time as demonstrated in Figure 13. The latter presents the reconstructed free oscillations of the shuttle after the manual application of a starting pulse. Despite the peak-to-peak vibration amplitude being larger than 40 μm; *i.e.*, 5 times the elementary grid pattern period, the continuity of the shuttle displacement is perfectly reconstructed thanks to the slightly different periods of the twin grids. The 1389.5 fps sampling rate allows for a high quality description of the 19.7 ms period of the shuttle vibration as observed in the zoom of Figure 13b. These performances give an idea of the method capabilities for real time processing. In this demonstration experiment, the limiting parameter is given by the image acquisition rate. Applied repeatedly to a single image kept in memory, the processing rate reaches 325 × 10^3^ Hz on this machine.

## 6. Software Package Description

In order to permit the reproducibility of results and the development of new applications, we provide the source code of the presented algorithms within a library called VERNIER along with the paper. The library is distributed under the GNU General Public License in the hope that it will be useful. The package can be downloaded from the website of MDPI (http://www.mdpi.com/) and from the website of the VERNIER project (http://www.femto-st.fr/vernier/).

While developing VERNIER, our goal was to allow a portable (independent from the hardware), fast, and reliable code. We also wanted to provide a package that is suitable for real-time implementation and that allows to obtain good performances with both simulations and real experiments from the same code. Therefore, we chose the C++ language for the implementation. VERNIER is built upon the cross-platform OpenCV library that provides a large compatibility with many image processing applications. OpenCV (http://opencv.org/) is a widely-used library of programming functions mainly aimed at real-time computer vision. VERNIER also uses FFTW routines to compute the discrete Fourier transforms. FFTW (http://www.fftw.org/) is one of the fastest libraries for computing the discrete Fourier transform in one or more dimensions [18].

VERNIER is written following C++ Coding Standards with the aim to be easily understandable and reusable. Describing the full implementation of the library is not the purpose of this article. For more information, a complete documentation is provided with the package.

## 7. Conclusions

The visual measurement of 1D displacements with a large range-to-resolution ratio as well as a high frequency rate suited for real time applications is presented and demonstrated in this paper. The subpixelic performances derive from phase computations applied to the images of periodic grids whereas the large unambiguous range is obtained thanks to twin stripe sets with slightly different periods. A 3-*σ* precision of 5 nm is demonstrated which has to be compared with the 168 μm measurement range. The image processing used is described in details and the source code is provided as supplementary materials to this paper. It involves neither Fourier transformation nor data fitting that are known to be time consuming. The demonstrated live processing rate is of 1,390 fps but the intrinsic method capabilities correspond to much higher rates once the limitations due to the image acquisition rate and to the operating system of the central processing unit are avoided.

The performance level can be adapted to the requirements of the final application in different ways. Firstly the method can be applied to the nanometer, the micrometer or the millimeter ranges (or even larger) by matching the actual values of the grid periods and of the vision system magnification accordingly. These conversion parameters from the object space domain to the image domain remains indeed ignored by the image processing routines that consider images only. Secondly as presented in the principle and numerical performance sections, the method resolution and unambiguous measurement range can be chosen almost independently from each other through the suitable choice of the grid period difference and of the camera signal-to-noise ratio and digitizing depth; provided that environmental disturbances remain sufficiently low. Thirdly, the vision system magnification does not have to be calibrated since the knowledge of the grid periods provides a size reference that is sufficient to convert pixels into actual distances in the reconstructed displacements.

These specifications make the method very attractive for a wide range of applications especially in robotics and automation. At present time a single direction is addressed and this point is one of the few limitations of the method. The extension of the proposed principle to measurements versus multi-degrees of freedom can however be envisaged for instance by using stereovison and/or multiple grid patterns. In this prospect, the capabilities of grid processing to comply with defocus is of particular interest [19].
